# Efficiency of DNA Mini-Barcoding to Assess Mislabeling in Commercial Fish Products in Italy: An Overview of the Last Decade

**DOI:** 10.3390/foods10071449

**Published:** 2021-06-22

**Authors:** Laura Filonzi, Marina Vaghi, Alessia Ardenghi, Pietro Maria Rontani, Andrea Voccia, Francesco Nonnis Marzano

**Affiliations:** Department of Chemistry, Life Sciences and Environmental Sustainability, University of Parma, Viale delle Scienze 11, 43124 Parma, Italy; laura.filonzi@unipr.it (L.F.); marina.vaghi@unipr.it (M.V.); aalessia.ardenghi@gmail.com (A.A.); pietromaria.rontani@unipr.it (P.M.R.); voccia.andrea@gmail.com (A.V.)

**Keywords:** food frauds, conservation, molecular genetics, mitochondrial DNA, cytochrome oxidase, biodiversity

## Abstract

The problem of fish traceability in processed products is still an important issue in food safety. Major attention is nowadays dedicated to consumer health and prevention of possible frauds regulated by national and international laws. For this reason, a technical approach is fundamental in revealing mislabeling at different levels. In particular, the use of genetic markers has been standardized and DNA barcoding is considered the gold-standard strategy to examine and prevent species substitution. Considering the richness of available DNA databases, it is nowadays possible to rapidly reach a reliable taxonomy at the species level. Among different approaches, an innovative method based on DNA mini barcoding has recently been proposed at an international level. Starting from this evidence, we herein illustrate an investigation dealing with the evolution of this topic in Italy over the last decade. The molecular analysis of 71 commercial fish samples based on mini-*COI* sequencing with two different primer sets reached an amplification success rate of 87.3 and 97.2%. The investigation revealed four major frauds (5.8%) and four minor ones (5.8%). Results highlighted a decrease in incorrect labeling in Italy from 32% to 11.6% over the last decade, although a recurrent involvement of “endangered” species *sensu* IUCN was still observed.

## 1. Introduction

The consumption of seafood products has increased all over the world during the last 50 years as demonstrated by data issued by the United Nations Food and Agriculture Organization (FAO) [1,2] that estimated the value of fish commerce to be over hundreds of billion dollars each year. Considering the importance of fish trade in the globalization era, consistent monitoring of the production chain focusing on technological developments, handling, processing and distribution by global networks is, therefore, necessary [3]. Nowadays, food quality and safety issues are crucial points for consumers, also considering the frequency of fish species substitutions. Basic consequences may be health problems that occur primarily through the consumption of cryptic species coming from contaminated areas or able to trigger allergy problems [4]. Despite financial fraud still being the main issue [5], major attention must be dedicated to such cryptic species as those belonging to the genera *Pangasius*, *Salmo* and *Tilapia* whose aquaculture exploitation makes them easy substitutes for wild species [4].

Precautionary measures are, therefore, necessary, particularly for products that are not visually recognizable at sight and are indistinguishable on a morphological basis after processing. Deliberate mislabeling and replacement of high-value species with cheaper ones is an Economically Motivated Adulteration (EMA) and is considered as fraud [5]. In a report paper published by the European Parliament in 2013, seafood was identified as the second most likely group of food to be subject to fraud, following olive oil [6].

Although the European Union labeling law (EU Regulation No. 1379/2013) [7] requests appropriate species traceability and labeling (scientific binomial nomenclature based on genus and species together with the common name), the identification of processed species is often difficult. Many scientists are working with innovative technologies to assess taxa identification and authenticity. Several molecular methods have been proposed to identify the correct species, from the use of single-protein or species-specific DNA sequences to modern genomic approaches. Among the wide variety of DNA methods nowadays available, the choice is mainly influenced by use simplicity and affordable costs in relation to the product value [8].

In recent years, the gold-standard strategy to examine and prevent species fraud has been DNA barcoding, a fast and cost-effective method for correct classification at a species level [9]. The original approach is based on the sequence analysis of a 650-bp mitochondrial DNA fragment. The cytochrome c oxidase I (*COI*) gene is the favorite sequence to act as a ‘‘barcode” to identify and delineate the animal lifeform [10]. Although the analysis of long DNA fragments is complex due to degradation along the different steps from production to analysis, most of the mtDNA-based studies have analyzed the full-length *COI* barcode [11,12]. Since the first applications of mitochondrial DNA barcoding [13], and then through constant methodological improvements [14], the most recent advances have led to an innovative approach based on mini-barcodes [15,16,17].

The methodology refers to the analysis of short DNA fragments and it can be applied in different fields of systematics (from museum collection research to forensic applications). *COI* mini-barcoding is, therefore, useful to assess the correct taxonomy in processed products. This is particularly the case with commercial fish, for which the correct preservation under stable refrigerated conditions is a major issue during fishing, transportation and distribution. In addition, fish processing for preservation is the major cause of DNA degradation. Consequently, molecular analyses to reveal species substitution may face DNA degradation limitations and, therefore, become biased by technical questions. The possibility of analyzing short DNA fragments such as *COI* mini-barcoding seems to solve this issue. In the field of ichthyology, the application of mini-barcoding is nowadays possible thanks to the availability of specific databases accounting for millions of *COI* sequences. To give a general idea, BOLD database reports cover more than 24,000 barcoded species among Actinopterygii and Elasmobranchii. On the other hand, it is noteworthy to observe that data redundancy may generate confusion or technical biases due to contradictory attribution within a genus level.

Starting from past experience of the application of *COI* and *CytB* barcoding, besides the use of additional markers [18], for the identification of species substitution in fish products [19], a new investigation was carried out to verify the usefulness of *COI* mini-barcoding as an innovative methodology to substitute for classic barcoding. The research was focused on sampling and analyzing the same taxa purchased in the same department stores to assess the evolution of cryptic species mislabeling after a decade, also considering the application of new European regulations [7]. The suitability of *COI* mini-barcoding and the evolution of the mislabeling issue are herein discussed, also considering some critical ecological, taxonomic and commercial aspects that have been arisen by experts based on their judgments.

## 2. Materials and Methods

Fresh and frozen commercial fish products were acquired in 10 different department stores located in the Emilia Romagna region during 2020 and 2021. The different department stores belonged to the major brands fully distributed over the entire country, with their own national fish provider. In this way, although executed locally, the research had national coverage.

A small sample of the edible tissue of approximately 50 mg was collected and fixed in absolute ethanol under refrigerated conditions. Samples were stored at −20 °C to be processed within one week of purchasing. A total number of 71 specimens belonging to 27 putative seawater species were analyzed. The samples dataset was prepared, listing the declared names and areas of fishing. The entire dataset is reported in Table 1. It is noteworthy to observe that four samples (MB43, MB64, MB66, MB71) did not report the origin in the label either as a specific FAO fishing area or a summary geographic declared one.

Genomic DNA was extracted and purified from about 10 mg of ethanol-preserved samples using the Wizard^®^ Genomic DNA Purification Kit (Promega, Madison, WI, USA). Purified DNA was evaluated by means of 1% agarose gel electrophoresis. No samples were discharged due to bad DNA quality although some of them were considered borderline (as outlined in the Results section).

Two different sets of primers were tested. The first pair of universal primers, Fish_miniFW: ATCACAAAGACATTGGCACCCT and Fish_miniRV: AATGAAGGGGGGAGGAGTCAGAA, specifically proposed by Sultana et al. [17] for fish species, were used and are herein named “Fish_Mini”. A 295 bp fragment of the mitochondrial *COI* gene was amplified through polymerase chain reaction (PCR) amplification using a Bio-Rad T100 Thermal Cycler. The cycling conditions were 95 °C for 10 min, followed by 34 cycles of 95 °C for 45 s, 57 °C for 45 s, 72 °C for 45 s and a final step at 72 °C for 10 min.

The second set of primers was tested following the protocol suggested by Shokralla et al. [15]. Amplicons of 226 base pairs were obtained using a primer pair originally called Mini_SH-E. The two primers, “Fish_miniE”_F 5′-CACGACGTTGTAAAACGACACYAAICAYAAAGAYATIGGCAC-3′ (forward) and Fish_miniE_R 5′-GGATAACAATTTCACACAGGCTTATRTTRTTTATICGIGGRAAIGC-3′ (reverse), were chosen and are herein referred to as “Fish_miniE”. The PCR was set as follows: 34 cycles of 45 s at 95 °C, 45 s at 67 °C, and 45 s at 72 °C, after an initial 10 min denaturation step at 95 °C and a final extension at 72 °C for 10 min.

The chemical conditions for both approaches were the following: a reaction volume of 20 µL—containing 1 U of GoTaq Polymerase (Promega, Madison, WI, USA), Mg^2+^ 1.5 mM, dNTPs 0.2 mM and 10 pmol of each primer—was used.

Amplicons were separated by 2.5% agarose gel electrophoresis and purified using the Qiagen MinElute PCR Purification Kit. The quality of the purified sample (1 μL) was visualized in 1.5% agarose gel that yielded clear bands. DNA quantity was evaluated using the Qubit dsDNA HS (High Sensitivity) Assay Kit (Invitrogen, Waltham, MA, USA) with a Qubit 3.0 Fluorometer (Life Technologies, Waltham, MA, USA). *COI* sequencing of both amplified regions was performed by the MACROGEN Europe service (Amsterdam, The Netherlands). Twenty percent of the samples were locally reanalyzed using a CEQ8000 DNA Analysis System (Beckman Coulter, Milan, Italy) based on capillary electrophoresis. The analytical conditions are reported in detail in Filonzi et al. [19].

The obtained sequences were manually corrected using MEGA 7.0 [20] and compared with those available in the genomic databases of GenBank using the BLAST service and BOLD (Barcode of Life Data System). In both cases, the species level was assigned when the identity rate was greater than 98% considering either BLAST or BOLD analyses [21]. The accession numbers of selected reference sequences displaying the highest identity score are available on request.

## 3. Results

Data concerning 71 analyzed samples are reported in Table 2. Considering the two different primer sets, “Fish_miniE” [15] successfully amplified 62 samples out of 71 (87.3% success rate) while “Fish_mini” [17] displayed a positive result in 69 of 71 samples (97.2%). In our dataset, four samples (MB28, MB29, MB66, MB71) did not have a clear scientific name (5.6%) and species comparison was executed based on common names. Similarly, four additional samples (MB43, MB64, MB66, MB71) did not report the origin on the label either as a specific FAO fishing area or a geographic declared region (5.6%).

The sequencing results and their comparison with available genomic databases allowed the recognition and identification of various fish species present in the analyzed commercial fish products. The obtained identity scores ranged between 96.5 and 100% in BLAST and 97.1 and 100% in BOLD. Mismatches between BLAST and BOLD were appropriately corrected by integrating either result. In fact, according to the 98% threshold value for species identification [21], three samples were discharged after using BLAST but all three of them were recovered after BOLD matching. Similarly, four products displayed a low identity score using BOLD but they were recovered using BLAST.

Among the 69 samples successfully attributed, a total number of eight species substitutions were detected (11.6%). In particular, four major frauds (MB19, MB25, MB38, MB44) related to mislabeling at the genus level (5.8%) and four minor (MB07, MB20, MB55, MB68) ones concerning different species within the same genus were detected (5.8%). The single names and substitutions are fully described in Table 2. Among the four major frauds, two samples labeled as *Katsowomus pelamis* (MB19 and MB25) turned out to be either *Thunnus thynnus* (GenBank) or *Thunnus maccoyii* (BOLD). Similarly, *Chelidonichtys lucerna* (BAR38) was found to be *Merluccius paradoxus* (GenBank and BOLD), and *Lamna nasus* (MB44) was *Ixurus oxyrinchus* (GenBank and BOLD). Concerning minor frauds, the four labeled species displayed lower identity percentages in BLAST and BOLD within the same genus: MB07 (respectively 97.1% and 99.1%), MB20 (96.4% and 97.5%), MB55 (97.1% for both databases), MB68 (97.5% and 97.1%).

Discrepancies between GenBank and BOLD were also evaluated and were evident in eight samples (MB07, MB19, MB20, MB25, MB37, MB49, MB50, MB59) (11.6%).

Interestingly, among the eight species substitutions, application of the IUCN index [22] revealed three Critically Endangered (CR) (MB19, MB25, MB44), two Near Threatened (NT) (MB55, MB68), two Least Concern (LC) (MB07, MB20) and one Not Evaluated (MB38) species.

## 4. Discussion

The mini-barcoding approach based on analysis of short *COI* fragments proved to be a useful methodology to define the correct taxonomy of commercial fish products. Among previously proposed primer sets, a choice was made based on the most reliable ones [15,17], and “Fish_miniE” showed a lower success rate than “Fish_mini”. In particular, the lower success of “Fish_miniE” (87.3%) was consistent with the one original report of 88.6% by Shokralla et al. [15], which was determined in a lower number of samples compared to our work. In fact, the authors correctly classified 39 of 44 samples, which is almost half the number of samples determined in our dataset. On the other hand, the experimental success of “Fish_mini” (97.2%) was in accordance with similar recent works (93.2%) based on longer sequences [23], and thus confirmed the reliability of the mini-barcoding methodology.

Although new markers are emerging for charismatic fish species and add technological improvements to this topic [24,25,26], the need for data-rich databases is still important, particularly in the case of widespread investigations at a national level. From this point of view, the BOLD database is considered better performing than BLAST; however, integration of different databanks is fundamental to reaching a reliable attribution. Consistency between databases should be expected but that is not always the case. On the other hand, a correct assessment at the species level is sometimes difficult due to contradictory identity scores and variable species names released by different databases. This aspect can generate trivial attributions, particularly whenever labeling does not report the scientific name or the appropriate fishing area, as happened in a limited number of our samples. Discrepancies between GenBank and BOLD had already emerged in the past and were evaluated by Sultana et al. [17], who evidenced that BOLD records were available for only 10,000 species. This number has recently rapidly increased to over 24,000 fish species; therefore, ambiguity problems should nowadays be limited to intraspecific variability and population diversity. Although intraspecific genetic diversity should be scarce using *COI*, the need for a continuous update of databases is important to reach a wide coverage of different populations over a large geographic scale.

In this research, the intervention of expert judgment was, therefore, necessary to evaluate genetic results that had to be integrated with sampling information useful to determine species-specific ecological characteristics (in particular, reported species name coupled to the area of origin, whenever available). To better clarify this concept, that was the case of sample MB19, which was labeled as Pacific *Katsuwonus pelamis*. BLAST and BOLD analyses returned two different Bluefin tuna species (*Thunnus thynnus* and *T. maccoyii*), therefore, revealing a mislabeled sample. Expert judgment was important in assigning the sample to Southern Bluefin Tuna rather than to Atlantic Bluefin Tuna according to both the identity score and the area of origin, the former species being a Pacific taxon consistent with the declared fishing zone.

Similarly, sample MB38 was not properly considered a fraud but rather hypothesized as an involuntary substitution that happened during delivery at the moment of purchasing. In fact, *Chelidonichtys lucerna* and *Merluccius paradoxus* have completely different meat colors and morphologies, even after processing, in their fillet appearance. Fillets of both species were also close to each other in the exposition counter. The product was classified anyway as a major fraud since the final delivery still belongs to the entire traceability process.

From a general point of view, the research evidenced 5.8% major frauds and 5.8% minor ones. Major and minor frauds were considered in relation to the taxonomic level of erroneous labeling. A final percentage of 11.6% of species substitutions were, therefore, discovered. It must be remarked that data are greatly variable on a world scale. As a matter of comparison, mislabeling was detected in 9.3% of seafood products in Germany [27], 24% in South Brazil [28] and 22% in India [29]. The results of Di Pinto et al. [30], based on molecular investigations, revealed an incredible occurrence of 82% of incorrect species declaration in fish fillet products. In some cases, the results could be biased by the product choice at the time of purchasing; this may be relevant whenever fillets are selectively chosen among suspected or evident frauds. In our investigation, fresh and frozen products were randomly bought with no particular attention to species or brands. Independent of local data, our results were in agreement with a recently published paper assuming that the most credible average mislabeling rate at the product level is 8% [31].

**Figure 1:**
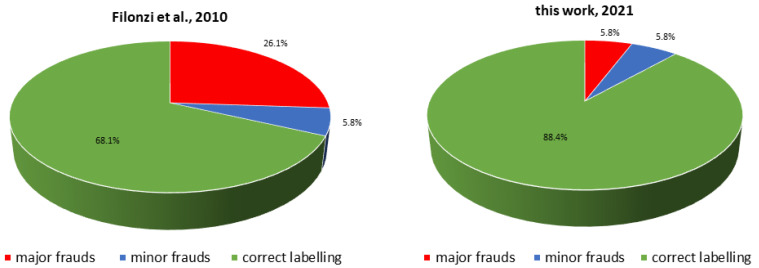
Comparison of percentages of species substitutions, both as major and minor frauds, assessed in 2010 and in the present work.

Besides the generalized assessment of multiple specimens, the experimental design considered a long time comparison starting from the previous work by Filonzi et al. [19] (see Figure 1). Our previous study [19] was among the first highlighting a high occurrence of incorrect species declaration in fillet fish products, underlying the strong trend towards seafood mislabeling in the Italian retail sector [30,32,33,34,35]. The past work [19] revealed incorrect labeling in 22 of 69 samples (32%), 18 of which were serious frauds (26.1%) from both the financial and nutritional points of view. The final aim of this new investigation was undoubtedly to assess the new trend after a decade of technical innovation and market surveillance. Results have evidenced a decrease in incorrect labeling in Italy from 32 to 11.6% over the last ten to eleven years. In particular, major frauds decreased from 26.1% to 5.8%. Data were in the low end of mislabeling rates reported in the literature [23]. It is noteworthy to observe that to minimize and prevent seafood frauds, proper regulations were issued in Europe and recommended on other continents. For example, fish labeling with both common and scientific names to be included on the product label together with FAO fishing area is nowadays mandatory and has been the current practice in Italy over the last decade. Similarly, Mariani et al. [36] also observed a sudden reduction of seafood mislabeling in Europe due to recent efforts in legislation governance with a positive impact on the entire commercial chain. Compared to data reported in this interesting publication [36], our percentages are in strict agreement with those obtained in several other European countries and fill the gap concerning the lack of data about Italy.

Nonetheless, another major issue is the illegal sacrifice and trade of endangered species widely protected by an international fishing ban. Fraudulent substitutions seem to continue, particularly in sharks and tunas, despite apparently increased control since the first published papers focusing on the problem [19,33]. Interestingly, application of the IUCN index [22] among eight species substitutions revealed three Critically Endangered (CR), two Near Threatened (NT), two Least Concern (LC) and one Not Evaluated species. This aspect is also related to conservation biology problems, rather than just the food safety system, and will have to be furtherly monitored using mini-barcoding, eventually widened to new genes [24]. In this respect Mini-barcoding could be considered as a sensitive application tool or applied to specific taxa highlighted as very often mislabeled and as leading to important health problems [37].

## 5. Conclusions

Mini-barcoding is a valuable tool to assess seafood species substitution, particularly in the case of processed products, displaying a high success rate. Despite DNA degradation, which may limit its diagnostic reliability, the majority of samples were correctly classified using BOLD and BLAST databases when supported by expert evaluation to solve cryptic nomenclature cases or apparent database redundancies. Although a general decreasing trend in fish species substitutions over the last decade was observed, consistent with similar trends in other European countries, a continuous update of datasets is important to reach a wide coverage of different species and populations. In particular, special attention should be focused on critically endangered species *sensu* IUCN whose involvement in mislabeling is recurrent and suggests still existing inappropriate control processes at different levels.

## Figures and Tables

**Table 1:** Detailed description of analyzed samples and information reported in the labels (common name, declared scientific name and fishing area). ‘‘N/A” (not available) refers to lacking label information.

Sample ID	Geographical Origin	Common Name	Declared Scientific Name
MB01	Southeast Atlantic Ocean	Deep-water Cape hake	*Merluccius capensis* e/o *M. paradoxus*
MB02	Northeast Atlantic Ocean	Pink salmon	*Oncorhynchus gorbuscha*
MB03	Northeast Atlantic Ocean—Iceland seabed	Atlantic cod	*Gadus morhua*
MB04	East Central Atlantic Ocean	Goldblotch grouper	*Epinephelus costae*
MB05	Northeast Atlantic Ocean	Pink salmon	*Oncorhynchus gorbuscha*
MB06	Eastern Indian Ocean	Swordfish	*Xiphias gladius*
MB07	East Central Atlantic Ocean	Angolan dentex	*Dentex angolensis*
MB08	Northeast Atlantic Ocean—Baltic Sea	Atlantic cod	*Gadus morhua*
MB09	Northeast Pacific Ocean	Pink salmon	*Oncorhynchus gorbuscha*
MB10	Northeast Atlantic Ocean—Baltic Sea	Atlantic cod	*Gadus morhua*
MB11	Southeast Atlantic Ocean	Blue shark	*Prionace glauca*
MB12	Northeast Atlantic Ocean	Haddock	*Melanogrammus aeglefinus*
MB13	Northeast Pacific Ocean	Pink salmon	*Oncorhynchus gorbuscha*
MB14	East Central Atlantic Ocean	Goldblotch grouper	*Epinephelus costae*
MB15	Northeast Atlantic Ocean	Haddock	*Melanogrammus aeglefinus*
MB16	Southeast Atlantic Ocean	Deep-water Cape hake	*Merluccius capensis* e/o *M. paradoxus*
MB17	Northeast Atlantic Ocean—Iceland seabed	Atlantic cod	*Gadus morhua*
MB18	Southeast Atlantic Ocean	Deep-water Cape hake	*Merluccius capensis* e/o *M. paradoxus*
MB19	Central Western Pacific Ocean	Skipjack tuna	*Katsuwonus pelamis*
MB20	East Central Atlantic Ocean	Angolan dentex	*Dentex angolensis*
MB21	Pacific Ocean Northeast or North West	Pink salmon	*Oncorhynchus gorbuscha*
MB22	Northwest Atlantic Ocean	Atlantic cod	*Gadus morhua*
MB23	Northeast Pacific Ocean	North Pacific hake	*Merluccius productus*
MB24	Southeast Atlantic Ocean	Swordfish	*Xiphias gladius*
MB25	Central Western Pacific Ocean	Skipjack tuna	*Katsuwonus pelamis*
MB26	Northeast Pacific Ocean	North Pacific hake	*Merluccius productus*
MB27	Northeast Atlantic Ocean—Iceland seabed	Atlantic cod	*Gadus morhua*
MB28	East Central Atlantic Ocean	Angolan dentex	N/A
MB29	Northeast Atlantic Ocean	Beaked redfish	N/A
MB30	Pacific Ocean Northeast or North West	Alaska pollock	*Theragra chalcogramma*
MB31	Northeast Atlantic Ocean	Atlantic cod	*Gadus morhua*
MB32	Pacific Ocean Northeast or North West	Pink salmon	*Oncorhynchus gorbuscha*
MB33	Pacific Ocean Northeast or North West	Alaska pollock	*Theragra chalcogramma*
MB34	Central or Southeast Pacific Ocean	Swordfish	*Xiphias gladius*
MB35	Southeast Atlantic Ocean	Blue shark	*Prionace glauca*
MB36	Southeast Atlantic Ocean	Swordfish	*Xiphias gladius*
MB37	Atlantic Ocean	Beaked redfish	*Sebastes norvegicus*
MB38	Northeast Atlantic Ocean	Tub gurnard	*Chelidonichtys lucerna*
MB39	East Central Atlantic Ocean	Spiny turbot	*Psettodes spp.*
MB40	East Central Atlantic Ocean	Swordfish	*Xiphias gladius*
MB41	Western Mediterranean	Leerfish	*Lichia amia*
MB42	Southeast Atlantic Ocean	Deep-water Cape hake	*Merluccius capensis* e/o *M. paradoxus*
MB43	N/A	Alaska pollock	*Theragra chalcogramma*
MB44	Northwest Atlantic Ocean	Porbeagle	*Lamna nasus*
MB45	Northeast Atlantic Ocean	European plaice	*Pleuronectes platessa*
MB46	Indian Ocean	Yellowfin tuna	*Thunnus albacares*
MB47	Lake Victoria	Nile perch	*Lates niloticus*
MB48	Northwest Atlantic Ocean	Picked dogfish	*Squalus acanthias*
MB49	Northeast Atlantic Ocean	Golden redfish	*Sebastes norvegicus*
MB50	East Central Atlantic Ocean	Bearded brotula	*Brotula barbata*
MB51	Northeast Atlantic Ocean	European plaice	*Pleuronectes platessa*
MB52	East Central Atlantic Ocean	Shortfin mako	*Isurus oxyrinchus*
MB53	Lake Victoria, Tanzania	Nile perch	*Lates niloticus*
MB54	East Central Atlantic Ocean	Porbeagle	*Isurus oxyrinchus*
MB55	North Sea	Smooth-hound	*Mustelus mustelus*
MB56	Northwest Atlantic Ocean	Halibut	*Reinhardtius hippoglossoides*
MB57	Unreported	Alaska pollock	*Theragra chalcogramma*
MB58	Northeast Atlantic Ocean	European plaice	*Pleuronectes platessa*
MB59	Iceland seabed	Beaked redfish	*Sebastes mentella*
MB60	Norwegian Sea	Saithe	*Pollachius virens*
MB61	Pacific Ocean Southwest	Shortfin mako	*Isurus oxyrinchus*
MB62	Norwegian Sea	Atlantic cod	*Gadus morhua*
MB63	Northeast Atlantic Ocean	Saithe	*Pollachius virens*
MB64	N/A	Swordfish	*Xiphias gladius*
MB65	Northeast Atlantic Ocean	European plaice	*Pleuronectes platessa*
MB66	N/A	Porbeagle	N/A
MB67	Northeast Atlantic Ocean	Atlantic cod	*Gadus morhua*
MB68	North Sea	Smooth-hound	*Mustelus mustelus*
MB69	Pacific Ocean Northwest	Pacific cod	*Gadus macrocephalus*
MB70	Northeast Atlantic Ocean	Atlantic cod	*Gadus morhua*
MB71	N/A	Alaska pollock	N/A

**Table 2:** Scientific names and identity percentage values of each sample are provided both for BLAST and BOLD alignment analysis. Major frauds: mislabeling at genus level; minor frauds: different species within the same genus. Major species substitutions in bold; minor substitutions underlined; N/A: Not Available.

Sample ID	Declared Scientific Name	GenBank Result (BLAST)	% Identity GenBank	BOLD Result	% Identity BOLD
MB01	*Merluccius capensis* e/o *M. paradoxus*	*Merluccius paradoxus*	99.1	*Merluccius paradoxus*	98.9
MB02	*Oncorhynchus gorbuscha*	*Oncorhynchus gorbuscha*	98.3	*Oncorhynchus gorbuscha*	97.7
MB03	*Gadus morhua*	*Gadus morhua*	98.6	*Gadus morhua*	98.4
MB04	*Epinephelus costae*	*Epinephelus costae*	98.3	*Epinephelus costae*	99.1
MB05	*Oncorhynchus gorbuscha*	*Oncorhynchus gorbuscha*	97.7	*Oncorhynchus gorbuscha*	98.0
MB06	*Xiphias gladius*	*Xiphias gladius*	100	*Xiphias gladius*	100
MB07	*Dentex angolensis*	*Dentex maroccanus*	99.1	*Dentex macrophtalmus*	100
MB08	*Gadus morhua*	*Gadus morhua*	98.3	*Gadus morhua*	98.1
MB09	*Oncorhynchus gorbuscha*	*Oncorhynchus gorbuscha*	98.6	*Oncorhynchus gorbuscha*	100
MB10	*Gadus morhua*	*Gadus morhua*	98.3	*Gadus morhua*	98.0
MB11	*Prionace glauca*	*Prionace glauca*	99.1	*Prionace glauca*	100
MB12	*Melanogrammus aeglefinus*	*Melanogrammus aeglefinus*	99.1	*Melanogrammus aeglefinus*	100
MB13	*Oncorhynchus gorbuscha*	*Oncorhynchus gorbuscha*	99.5	*Oncorhynchus gorbuscha*	100
MB14	*Epinephelus costae*	*Epinephelus costae*	98.2	*Epinephelus costae*	99.1
MB15	*Melanogrammus aeglefinus*	*Melanogrammus aeglefinus*	99.5	*Melanogrammus aeglefinus*	100
MB16	*Merluccius capensis* e/o *M. paradoxus*	*Merluccius paradoxus*	98.2	*Merluccius paradoxus*	99.1
MB17	*Gadus morhua*	*Gadus morhua*	98.2	*Gadus morhua*	100
MB18	*Merluccius capensis* e/o *M. paradoxus*	*Merluccius paradoxus*	100	*Merluccius paradoxus*	100
MB19	*Katsuwonus pelamis*	***Thunnus thynnus***	98.7	***Thunnus maccoyii***	100
MB20	*Dentex angolensis*	*Dentex maroccanus*	96.9	*Dentex macrophtalmus*	99.4
MB21	*Oncorhynchus gorbuscha*	*Oncorhynchus gorbuscha*	98.8	*Oncorhynchus gorbuscha*	100
MB22	*Gadus morhua*	*Gadus morhua*	99.1	*Gadus morhua*	98.7
MB23	*Merluccius productus*	*Merluccius productus*	98.8	*Merluccius productus*	100
MB24	*Xiphias gladius*	*Xiphias gladius*	98.7	*Xiphias gladius*	100
MB25	*Katsuwonus pelamis*	***Thunnus thynnus***	99.2	***Thunnus maccoyii***	100
MB26	*Merluccius productus*	*Merluccius productus*	99.1	*Merluccius productus*	100
MB27	*Gadus morhua*	*Gadus morhua*	99.1	*Gadus morhua*	100
MB28	N/A	*Dentex angolenses*	99.1	*Dentex angolenses*	100
MB29	N/A	*Sebastes mentella*	99.1	*Sebastes mentella*	99.5
MB30	*Theragra chalcogramma*	*Theragra chalcogramma*	99.5	*Gadus chalcogrammus*	100
MB31	*Gadus morhua*	*Gadus morhua*	99.4	*Gadus morhua*	98.8
MB32	*Oncorhynchus gorbuscha*	*Oncorhynchus gorbuscha*	99.5	*Oncorhynchus gorbuscha*	100
MB33	*Theragra chalcogramma*	*Theragra chalcogramma*	99.5	*Gadus chalcogrammus*	100
MB34	*Xiphias gladius*	*Xiphias gladius*	99.5	*Xiphias gladius*	100
MB35	*Prionace glauca*	*Prionace glauca*	99.5	*Prionace glauca*	99.4
MB36	*Xiphias gladius*	*Xiphias gladius*	100	*Xiphias gladius*	100
MB37	*Sebastes norvegicus*	*Sebastes norvegicus*	99.1	*Sebastes viviparus*	100
MB38	*Chelidonichtys lucerna*	***Merluccius paradoxus***	98.7	***Merluccius paradoxus***	99.1
MB39	*Psettodes spp.*	*Psettodes bennettii*	99.1	*Psettodes bennettii*	99.5
MB40	*Xiphias gladius*	*Xiphias gladius*	100	*Xiphias gladius*	100
MB41	*Lichia amia*	*Lichia amia*	99.1	*Lichia amia*	99.0
MB42	*Merluccius capensis* e/o *M. paradoxus*	*Merluccius paradoxus*	99.1	*Merluccius paradoxus*	98.1
MB43	*Theragra chalcogramma*	*Theragra chalcogramma*	99.1	*Gadus chalcogrammus*	100
MB44	*Lamna nasus*	***Isurus oxyrinchus***	99.5	***Isurus oxyrinchus***	99.5
MB45	*Pleuronectes platessa*	*Pleuronectes platessa*	100	*Pleuronectes platessa*	99.1
MB46	*Thunnus albacares*	*Thunnus albacares*	99.6	*Thunnus albacares*	99.1
MB47	*Lates niloticus*	*Lates niloticus*	100	*Lates niloticus*	100
MB48	*Squalus acanthias*	*Squalus acanthias*	99.1	*Squalus acanthias*	98.2
MB49	*Sebastes norvegicus*	*Sebastes norvegicus*	99.1	*Sebastes viviparus*	99.5
MB50	*Brotula barbata*	*Brotula barbata*	98.3	*Brotula multibarbata*	99.5
MB51	*Pleuronectes platessa*	*Pleuronectes platessa*	99.1	*Pleuronectes platessa*	99.5
MB52	*Isurus oxyrinchus*	*Isurus oxyrinchus*	99.2	*Isurus oxyrinchus*	99.5
MB53	*Lates niloticus*	*Lates niloticus*	98.2	*Lates niloticus*	97.1
MB54	*Isurus oxyrinchus*	*Isurus oxyrinchus*	99.5	*Isurus oxyrinchus*	99.5
MB55	*Mustelus mustelus*	*Mustelus asterias*	98.3	*Mustelus asterias*	98.3
MB56	*Reinhardtius hippoglossoides*	*Reinhardtius hippoglossoides*	99.1	*Reinhardtius hippoglossoides*	99.5
MB57	*Theragra chalcogramma*	*Theragra chalcogramma*	98.7	*Gadus chalcogrammus*	99.4
MB58	*Pleuronectes platessa*	*Pleuronectes platessa*	98.3	*Pleuronectes platessa*	99.1
MB59	*Sebastes mentella*	*Sebastes mentella*	99.1	*Sebastes viviparus*	99.5
MB60	*Pollachius virens*	*Pollachius virens*	99.1	*Pollachius virens*	100
MB61	*Isurus oxyrinchus*	*Isurus oxyrinchus*	99.1	*Isurus oxyrinchus*	99.4
MB62	*Gadus morhua*	*Gadus morhua*	99.5	*Gadus morhua*	99.5
MB63	*Pollachius virens*	*Pollachius virens*	99.5	*Pollachius virens*	100
MB64	*Xiphias gladius*	*Xiphias gladius*	98.8	*Xiphias gladius*	100
MB65	*Pleuronectes platessa*	*Pleuronectes platessa*	99.1	*Pleuronectes platessa*	99.5
MB66	N/A	N/A	N/A	N/A	N/A
MB67	*Gadus morhua*	*Gadus morhua*	96.5	*Gadus morhua*	98.6
MB68	*Mustelus mustelus*	*Mustelus asterias*	98.1	*Mustelus asterias*	97.5
MB69	*Gadus macrocephalus*	N/A	N/A	N/A	N/A
MB70	*Gadus morhua*	*Gadus morhua*	99.1	*Gadus morhua*	97.8
MB71	N/A	*Theragra chalcogramma*	99.1	*Gadus chalcogrammus*	98.3
